# Viral Vector Based Immunotherapy for Peanut Allergy

**DOI:** 10.3390/v16071125

**Published:** 2024-07-13

**Authors:** Miguel Gonzalez-Visiedo, Roland W. Herzog, Maite Munoz-Melero, Sophia A. Blessinger, Joan M. Cook-Mills, Henry Daniell, David M. Markusic

**Affiliations:** 1 Department of Pediatrics, Herman B Wells Center for Pediatric Research, Indiana University School of Medicine, Indianapolis, IN 46202, USAmamelero@iu.edu (M.M.-M.); soabless@iu.edu (S.A.B.); joancook@iu.edu (J.M.C.-M.);; 2 Department of Basic and Translational Sciences, School of Dental Medicine, University of Pennsylvania, Philadelphia, PA 19104, USA; hdaniell@upenn.edu

**Keywords:** adeno-associated virus, liver gene transfer, immune tolerance, food allergy, peanut allergy

## Abstract

Food allergy (FA) is estimated to impact up to 10% of the population and is a growing health concern. FA results from a failure in the mucosal immune system to establish or maintain immunological tolerance to innocuous dietary antigens, IgE production, and the release of histamine and other mediators upon exposure to a food allergen. Of the different FAs, peanut allergy has the highest incidence of severe allergic responses, including systemic anaphylaxis. Despite the recent FDA approval of peanut oral immunotherapy and other investigational immunotherapies, a loss of protection following cessation of therapy can occur, suggesting that these therapies do not address the underlying immune response driving FA. Our lab has shown that liver-directed gene therapy with an adeno-associated virus (AAV) vector induces transgene product-specific regulatory T cells (Tregs), eradicates pre-existing pathogenic antibodies, and protects against anaphylaxis in several models, including ovalbumin induced FA. In an epicutaneous peanut allergy mouse model, the hepatic AAV co-expression of four peanut antigens Ara h1, Ara h2, Ara h3, and Ara h6 together or the single expression of Ara h3 prevented the development of a peanut allergy. Since FA patients show a reduction in Treg numbers and/or function, we believe our approach may address this unmet need.

## 1. Introduction

Approximately two decades ago, liver-directed gene therapy with adeno-associated viral (AAV) vectors emerged as a method of immune tolerance induction [1,2]. Since then, multiple studies have found that gene products expressed from AAV vectors in hepatocytes can induce antigen-specific tolerance [3]. In other works, the hepatocyte or liver sinusoidal endothelial cell-restricted expression from lentiviral vectors was shown to have similar tolerogenic capabilities [4,5,6]. Initial studies focused on the expression of therapeutic proteins that are used in gene and protein replacement therapies to treat genetic diseases, such as hemophilia [1,7] or lysosomal storage disorders [8]. In these studies, hepatic immune tolerance induction prevented the rejection of the gene therapy, allowed for gene transfer to extrahepatic sites, and prevented anti-drug antibody formation against intravenous protein replacement therapy. Furthermore, a reversal of pre-existing antibody production was possible [9,10]. Moreover, reversal of a neutralizing antibody against coagulation factor VIII was demonstrated in a hemophilia A patient in an ongoing clinical trial, providing the first evidence of immune tolerance induction by hepatic AAV gene therapy in humans [11]. In recent years, research on several pre-clinical models has shown that this concept is also applicable to the treatment of autoimmune diseases and severe allergies [12,13,14,15]. 

The liver micro-environment is characterized by immune suppressive properties, which are thought to have evolved from the need to prevent inflammation induced by ingested antigens that reach the liver via the gut–liver axis [16]. An unfortunate consequence of this immunological hypo-responsiveness includes the persistence of hepatitis viruses and inadequate responses to liver tumors. However, peripheral tolerance induction via hepatic gene transfer may be exploitable for therapeutic purposes. A combination of high levels of immune suppressive cytokines such as IL-10 and TGF-β, a network of professional and non-professional antigen presenting cells, the expression of checkpoint molecules such as PD-1, and the ability to induce FoxP3^+^ regulatory CD4^+^ T cells (Treg) in the liver and hepatic lymph nodes tends to induce immune tolerance if sufficient levels of antigen are expressed [3]. The deletion of effector T cells and Treg induction are required, as they work in concert to tilt the response toward tolerance [1]. The rapid egression of hepatic induced Treg enforces tolerance systemically [17]. Treg are also required for the maintenance of tolerance, and the frequencies of induced Treg correlate with the levels of antigen expression [10]. In addition, high antigen levels may directly inhibit memory B cells and elevate IL-10 expression in the liver micro-environment, which is critical for the suppression of cytotoxic CD8^+^ T cell responses [10,18]. 

Allergies result from a damaging immune response against substances (“allergens”) in the environment that are harmless to most people. In its most severe form, the allergic response can become dangerous, e.g., result in anaphylaxis, a medical emergency that represents a life-threatening acute hypersensitivity reaction. People can be sensitized to allergens by oral or respiratory routes or via skin exposure. Food allergies are common in many human populations. For example, it is estimated, based on data from the U.S. Centers for Disease Control and Prevention, that in the United States approximately 20 million Americans were affected in 2021, including 4 million children (allergyasthmanetwork.org). From an immunology point of view, an allergy is an aberrant immune response resulting from disruption in the normal tolerance induction pathways, either through mutations in endogenous proteins or exposure to an allergen under proinflammatory conditions. A lack or breakdown of oral tolerance skews the immune response towards Th2, resulting in the production of IL-4 and IL-13 cytokines by T helper cells and ultimately the production of IgE [19]. Anaphylactic reactions can be triggered upon IgE binding to an allergen and the crosslinking of high affinity FceRI receptors on immune cells, such as mast cells and basophils, resulting in the release of proinflammatory molecules. Since allergy induction and maintenance is dependent on Th2 immunity, one approach to treat allergy may lie in the induction of a functional Treg response against the allergen [20,21,22]. Oral immunotherapy (OIT) and sublingual immunotherapy (SLIT) approaches have been developed to desensitize patients by a careful dosing of the allergen over time [23]. These therapies require frequent repeated allergen administration. However, in addition to compliance issues, this approach may not result in durable tolerance unless initiated early in life [24,25]. A later initiation of OIT tends to transiently direct immune deviation away from IgE toward IgG formation rather than shut down antibody formation. 

Hepatic AAV gene transfer has resulted in long-lasting immune tolerance to various transgene products in adult murine and canine models, preventing both IgG and IgE formation against the protein antigen [3,26,27,28]. In our previous work in an animal model of an egg allergy, we successfully treated mice that had been epicutaneously sensitized to chicken ovalbumin by hepatic AAV8 gene transfer [13]. A single-dose administration of an AAV8 vector expressing the whole antigen completely protected the mice from allergy sensitization and, upon further optimization, was also effective in animals with an established allergy. Here, we sought to adopt this approach to the treatment of peanut allergy, as most of the cases of food allergy-related anaphylaxis result from peanut allergy [29,30,31]. Peanut allergy is among the most common food allergies in the US (affecting 1.8% of the population) and, in contrast to egg or milk allergies, is more likely to last lifelong [32,33]. A drug based on peanut flour for OIT recently received regulatory approval in the US [34,35,36,37,38]. Multiple allergens contained in peanuts have been shown to contribute to the allergic response, making it a more challenging target for a gene therapy approach [39,40]. Given the limited packaging capacity of AAV vectors of ~5 kb, we developed a series of vectors, each expressing a single peanut allergen. Individual vectors or a vector cocktail were tested in a mouse model of cutaneous peanut sensitization. In contrast to single allergen vectors, the vector cocktail (expressing a total of four peanut allergens) was highly effective in preventing antibody formation and clinical allergy symptoms. Unexpectedly however, using a higher dose of a single allergen (Ara h3) was nearly as effective. 

## 2. Materials and Methods

### 2.1. Construction of Plasmids

Ara h1 (P43237), Ara h2 (ACN62248.1), Ara h3 (ABI17154), and Ara h6 (Q647G9) sequences were obtained from UniProt with their respective ascension numbers in parentheses. Sequences were synthesized and subcloned into either a single-stranded AAV (ssAAV) backbone [41] (Ara h1 and Ara h3) or self-complementary (scAAV) backbone [15] (Ara h2 and Ara h6) with a hepatocyte specific ApoE enhancer and hAAT (human α_1_-antitrypsin) promoter, and a synthetic intron. 

### 2.2. AAV Production

ApoE-hAAT-Ara h1, ApoE-hAAT-Ara h2, ApoE-hAAT-Ara h3, ApoE-hAAT-Ara h6, and ApoE-hAAT-FIX AAV8 vectors were produced by the transfection of HEK-293 cells and purified by iodixanol gradient as published [42]. 

### 2.3. Experimental Mice 

C57BL/6J mice were purchased from Jackson Laboratories (Bar Harbor, ME, USA). Flaky tail mice with homozygous mutations in FLg^ft/ft^/Tmem79^ma/ma^ (FT^−/−^) were kindly donated by Dr. Joan Cook-Mills. Female FT^+/−^ mice were generated by breeding FT^−/−^ male mice with C57BL/6J females as described [43,44]. Mice were genotyped using a protocol as described by Walker et al. [45]. These studies were approved on 1 Feb 2022 under protocol 21179 by the Institutional Animal Care and Use Committee (IACUC) of Indiana University School of Medicine.

### 2.4. Peanut Extract Protocol

We mixed grounded unsalted roasted peanuts 1:5 with PBS containing NaCl at 1M at pH 8.5 for 2 h at RT. A soluble fraction was collected after centrifugation at 10,000× *g* for 45 min at 4 °C. Supernatant was sterilized with passage through a 0.2 mm filter, and protein concentration were determined by Bradford assay [46,47]. 

### 2.5. Qualitative and Quantitative Comparison of Peanut Extracts

Twenty μg of total protein of peanut extract was resuspended on loading buffer containing B-ME as a denaturalizing agent and incubated at 95 degrees for 5 min. Samples were loaded on a pre-stained polyacrylamide gel from Bio-Rad (Hercules, CA, USA). Gel image was captured on a Bio-Rad ChemiDoc MP Imaging System. The levels of Ara h1, Ara h2, Ara h3, and Ara h6 proteins were measured using Ara h specific ELISA kits (ARA H ELISA 2.0 kits) from Indoor Biotech (Charlottesville, VA, EEUU, USA) following manufacturer’s instructions. 

### 2.6. Animal Treatment

For the FA prevention studies, 6-week-old female FT^+/−^ mice were IV injected with 1 × 10^11^ vg of a single or a cocktail of all four vectors (1 × 10^11^/vector) per mouse of ApoE-hAAT-Arah1, ApoE-hAAT-Arah2, ApoE-hAAT-Arah3, ApoE-hAAT-Arah6 in a total volume of 200 mL in sterile PBS. Mice were bled from the retro-orbital plexus 4 weeks after AAV injection under anesthesia using heparinized capillary tubes. Then, mice were sensitized based on Walker et al. with 20 mg of Alternaria alternata extract (Greer Laboratories, Lenoir, NC, EEUU, USA) followed by 200 mg of peanut extract [45]. This procedure was repeated on days 0, 3, 6, 9, 12, 14, 16, and 18. FT^+/−^ mice were bled as previously described on day 19 and challenged through IP injection of 1 mg of peanut extract. Some mice received a second challenge with peanut extract four weeks following the first challenge.

### 2.7. Challenge Procedure

FT ^+/−^ female mice were challenged by IP injection of 1 mg of peanut extract diluted in 200 mL of sterile PBS. Core body temperature was recorded before and 15, 30, and 45 min after PE administration with a rectal thermometer (Physitemp Instruments, Clifton, NJ, EEUU, USA). The phenotype following the challenge was recorded using a symptom scale (Table 1). 

### 2.8. Vector Copy Numbers and mRNA Levels in Liver Tissue

Vector genome copy numbers (VGCN) were determined using digital droplet PCR (ddPCR) using the following primers and EvaGreen Supermix. cDNA from mRNA was generated with Bio-RAD cDNA kit. The expression of mRNA was normalized to beta-actin expression, and vector copy number and mRNA expression in the liver were calculated as previously described using transgene specific primer pairs [48]. Primers were as follows: Ara h1: FWD: 5′-CCGAGACCAATCATCCTACTTG-3′ and REV: 5′-CTCCTCCTGCATTCTCTTCTAAC-3′; Ara h2: FWD: 5′-CCAACGTGACGAGGATTCAT-3′ and REV: 5′-TCTCCGATCATATGGACTAGGG-3′; Ara h3: FWD: 5′-GCCCAGATGAAGAAGATGAGAG-3′ and REV: 5′-CACGTTGTTGTGGCCTAGAT-3′; Ara h6: FWD: 5′-GAGCAGTACGACTCCTACGATA-3′ and REV: 5′-CATCTCTGTGTGTTCTCCATCTC-3′.

### 2.9. Liver Lysates Preparation

Liver was collected from FT^+/−^ mice 4 weeks after AAV administration. Livers were homogenized in lysis buffer using a Bead Mill 24 (Thermo Fisher Scientific, Waltham, MA, EEUU, USA) and protein lysates were obtained using the Qproteome Mammalian Protein Prep Kit (Qiagen, Germantown, MD, EEUU, USA) following the manufacturer’s instructions. Protein content was determined using a Bradford assay (Bio-Rad, Hercules, CA, EEUU, USA). The levels of Ara h1, Ara h2, Ara h3, and Ara h6 proteins were measured using an ELISA kit from Indoor Biotech following manufacturer’s instructions.

### 2.10. Analysis of Plasma Samples

PE-specific IgG1 titers were determined in plasma by ELISA based on Biswas et al. IgE levels were determined using an Anti-PE (PE extract or using purified Ara h1, Ara h2, Ara h3, or Ara h6 proteins) as described by Walker et al. [45]. The levels of Ara h1, Ara h2, Ara h3, and Ara h6 proteins in mouse plasma and liver lysates were quantified using a peanut antigen specific ELISA (Indoor Biotech) following manufacturer’s instructions.

## 3. Results

Food allergies may result from initial exposure on the skin, either under inflammatory conditions such as atopic dermatitis or in patients with a compromised skin barrier resulting from genetic mutations in critical proteins for barrier maintenance, before oral tolerance is achieved [25]. To generate a murine model of skin sensitization, mutations in two proteins involved in skin barrier defense against pathogens and allergens (filaggrin and mattrin) were introduced in the flaky tail mouse model [45]. Heterozygous female FT^+/−^ mice responded to the allergens administered via the epicutaneous route. Here, we sought to use this model to test for the ability of hepatic AAV gene transfer to protect from peanut allergy. We chose to express four common peanut allergens as a transgene and evaluated their ability to protect against a peanut allergy based on the reported incidence of IgE reactivity in human patients [39,40,49,50,51,52]. 

### 3.1. Characterization of Peanut Extract 

**Figure 1:**
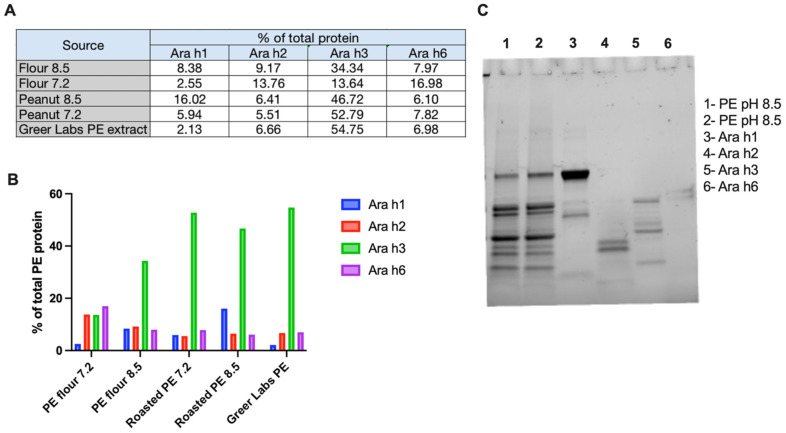
Characterization of in-house generated peanut extract. (**A**) Raw values of relative Ara h1, Ara h2, Ara h3, and Ara h6 levels measured by Ara h specific ELISA (Indoor Biotechnologies) in peanut extracts from peanut flour and dry roasted peanuts at different pHs (7.2 and 8.5) and a commercial peanut extract (Stallergenes Greer). (**B**) Relative abundance of Ara h1, 2, 3, and 6 in peanut extracts obtained from different sources. (**C**) SDS-PAGE of two different peanut protein extracts and purified Ara h1, 2, 3, and 6 peanut proteins (Indoor Biotechnologies).

To generate a peanut extract for sensitization, we followed a published protocol evaluating two different pHs for our peanut extraction buffer (pH 7.2 and 8.5) and two different source materials (peanut flour or dry roasted peanuts) [53]. Following concentration by molecular weight cutoff centrifugation, we determined total protein by Bradford assay and the levels of Ara h1, Ara h2, Ara h3, and Ara h6 antigens using an antigen specific ELISA (Figure 1A,B). Overall, we detected lower levels of the four specific Ara h proteins in extracts from peanut flour compared to roast peanuts. In most of the extracts, Ara h3 was the most abundant of the four peanut allergens, in line with what has been reported in the literature [54]. Extracts from roasted peanuts more closely resembled the makeup of the commercial peanut extract from Stallergenes Greer labs. To characterize the reproducibility of our extraction protocol, we ran two separate extractions from dry roasted peanuts on a pre-stained SDS-PAGE gel, along with purified Ara h1, Ara h2, Ara h3, and Ara h6. The banding patterns of the two extracts were similar, and we observed representative bands in our extracts when compared to the purified Ara h proteins (Figure 1C). Thus, we elected to use the roasted peanut extracted in pH 8.5 buffer for our studies. Because these four Ara h proteins constituted approximately 70–75% of the total protein content in the peanut extract, and the anti-IgE antibodies against these Ara h proteins are associated with peanut allergy in patients, we selected these four Ara h proteins to be expressed from AAV vectors.

**Figure 2:**
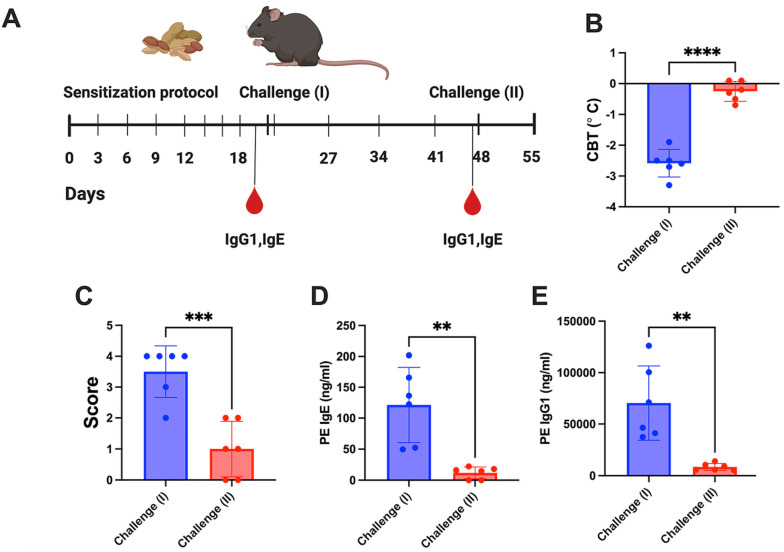
Validation of peanut allergy sensitization in the FT^+/−^ model and assessment of allergic responses following two separate challenges. (**A**) Experimental timeline followed. (**B**) Changes in core body temperature from baseline to 30 min post challenge. (**C**) Symptom score (see Table 1 for definitions) following challenge with peanut extract after sensitization and four weeks later. (**D**) Peanut-specific levels of IgE and (**E**) IgG1 measured after peanut sensitization and four weeks later. Data are presented as single data points and means ± standard deviation. Statistical testing was conducting using unpaired T-test for all the data sets excluding panel (**C**), which was performed using the Mann–Whitney test. ** *p* < 0.01, *** *p* > 0.001 and **** *p* < 0.0001.

Next, we determined if we could trigger an allergic response to peanuts in our animal model (Figure 2A). Previous work in newborn mice showed that these animals could be sensitized with both ovalbumin (ova) and peanut extract, and that sensitized animals developed moderate anaphylactic responses following oral challenge with the respective allergens [45]. However, when we tested this in adult female FT^+/−^ mice, both ova and peanut extract sensitized adult mice required an IP challenge to trigger an allergic response [13]. Of note, the severity of the allergic response, as measured by the changes in core body temperature post challenge (Figure 2B) and the symptom score (Figure 2C), was less severe to peanut extract when compared to ova. An analysis of independent sensitization studies showed that all animals developed measurable IgE and IgG titers to whole peanut extract (Figure 2D,E). We next tested if peanut sensitized mice would respond to a delayed challenge (challenge II), four weeks post sensitization, to evaluate our therapy in animals with an established allergy. However, unlike ovalbumin, a four-week delay in peanut extract challenge in peanut sensitized FT^+/−^ mice did not develop anaphylaxis (Figure 2B) and had a mild allergic response (Figure 2C). This lack of response was associated with a loss in circulating IgE and IgG titers (Figure 2D,E). 

### 3.2. Hepatic Gene Transfer with a Cocktail of Vectors Expressing 4 Allergens Protects from Peanut Allergy while Single Vectors Are Ineffective

**Figure 3:**
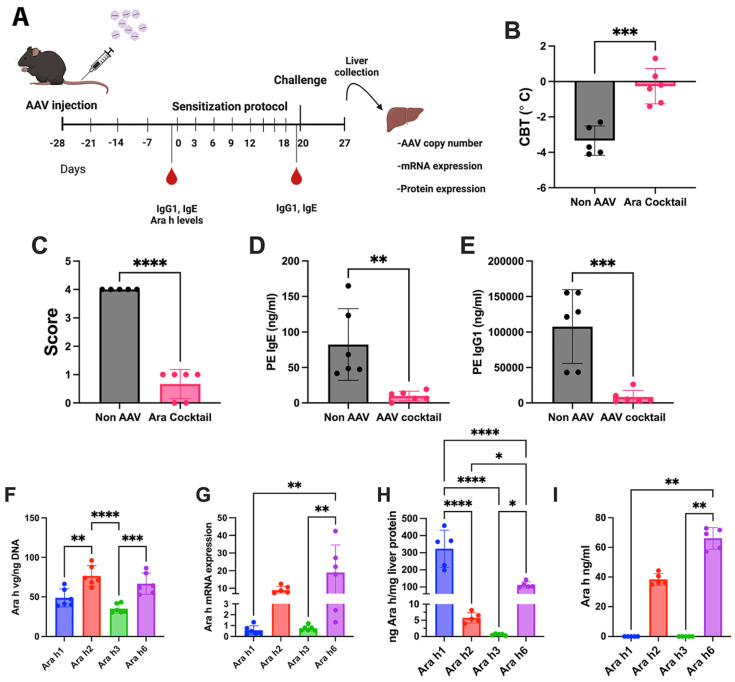
AAV Ara h cocktail prevents peanut sensitization. (**A**) Experimental timeline followed. (**B**) Changes in core body temperature and (**C**) symptom score (see Table 1 for definitions) following challenge with peanut extract. (**D**) Peanut-specific levels of IgE and (**E**) IgG1. (**F**) Ara h vector genome (vg) copy numbers and (**G**) Ara h mRNA expression in liver samples. (**H**) Expression of Ara h proteins in liver and (**I**) plasma. Data are presented as single data points and means ± standard deviation. Statistical testing was conducting using unpaired T-test for panels (**B**–**E**), one-way ANOVA statistic test was used for panels (**F–H**) and Kruskal–Wallis test was used for panel (**I**). * *p* < 0.05, ** *p* < 0.01, *** *p* < 0.001 and **** *p* < 0.0001.

AAV serotype 8 vectors expressing Ara h1, Ara h2, Ara h3, or Ara h6 antigens from a hepatocyte-specific enhancer/promoter (ApoE/human α_1_-antitrypsin) were generated (Supplementary Figure S1) [1,55]. FT^+/−^ mice (6-week-old female animals, n = 6/experimental group) received hepatic gene transfer using a dose of 1 × 10^11^ vg/vector. An initial study was conducted in which FT^+/−^ mice were transduced with a mixture of all 4 vectors (1 × 10^11^ vg of each vector). One month after vector administration, animals were sensitized to peanuts with a 3-week skin sensitization regimen (epicutaneous administration of peanut extract with Alternaria extract as adjuvant), followed by a single intraperitoneal challenge with peanut protein extract, as outlined in Figure 3A. Mice that were not transduced with a vector served as the positive control for antibody formation and allergic reaction. Control animals developed anaphylaxis and had a moderate allergic response, as measured by a change in core body temperature and peanut allergy score following challenge with peanut extract (Figure 3B,C) and had IgE and IgG antibodies reactive to the peanut extract (Figure 3D,E). Importantly, animals that were pre-treated with the cocktail of the four AAV8-Ara h vectors were protected from anaphylaxis, had mild allergic symptoms (Figure 3B,C) and had a significant reduction in peanut reactive IgE and IgG antibodies (Figure 3D,E). At the end of the study, livers were collected to determine the vector genome copy numbers of the respective vectors, the mRNA levels of the respective Ara h genes, and the protein levels of the respective of the Ara h proteins (Figure 3F–H). In addition to the liver, the plasma levels of the Ara h proteins were also measured by ELISA (Figure 3I). In the animals receiving the cocktail of AAV8-Ara h vectors, we observed significantly higher copy numbers of AAV8-Ara h2 and AAV8-Ara h6 vectors in the liver; however, the overall copy numbers were in a comparable range, suggesting that the total dose of 4 × 10^11^ vgs was not saturating for AAV receptor-mediated uptake in the liver (Figure 3F). In contrast, we observed differential mRNA levels of each of the Ara h genes by qPCR, with a higher mRNA expression of Ara h2 and Ara h6 (Figure 3G). This may be in part due to the differences in vector construction, as Ara h2 and Ara h6 were packaged as a self-complementary AAV (scAAV) vectors, due to their smaller size, whereas Ara h1 and Ara h3 were packaged as single-stranded AAV (ssAAV) vectors [56]. However, when measuring the levels of expressed antigen in liver extracts and in the plasma, we observed an opposite effect (Figure 3H). Antigen levels in the liver were highest for Ara h1, followed by Ara h6, Ara h2, and Ara h3 (which was >3 logs lower compared to Ara h1). Ara h2 and Ara h6 antigens were also found in plasma, while Ara h1 and Ara h3 were absent in the circulation (Figure 3I). The high ratio of antigen in plasma compared to the liver suggests that Ara h2 and Ara h6 are efficiently secreted by hepatocytes, whereas Ara h1 and h3 likely lack an efficient secretion signal in mammals. It should be noted that no codon optimization was employed for the expression of these proteins in mammalian cells, and this may have contributed to differences in translation efficiency. As expected, no signal was detected for vg copy number, Ara h mRNA, and Ara h proteins in the liver and plasma of control mice. 

**Figure 4:**
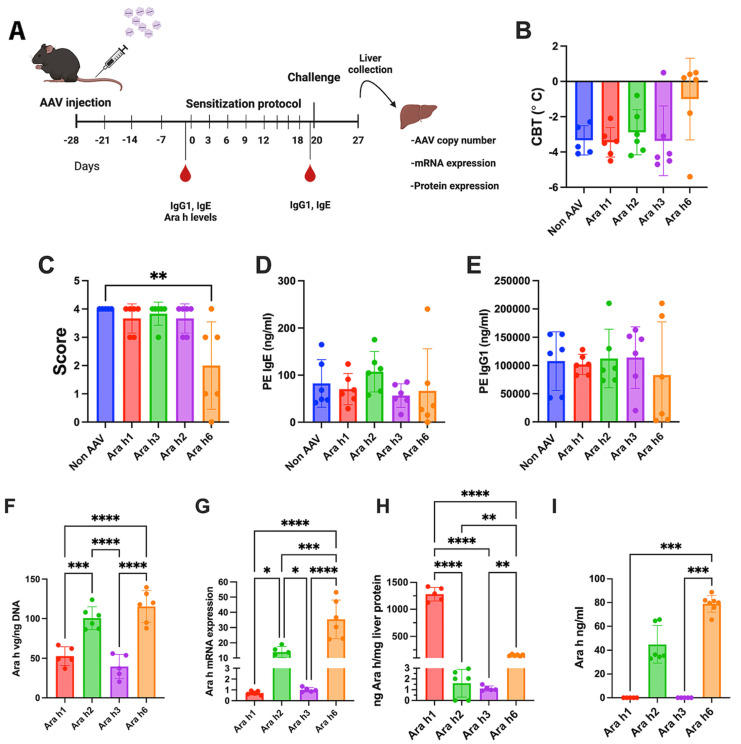
Single AAV-Ara h vectors are less effective for PE prophylaxis. (**A**) Experimental timeline followed. (**B**) Changes in core body temperature and (**C**) symptom score (refer to symptom score table here) following challenge with peanut extract. (**D**) Peanut-specific levels of IgE and (**E**) IgG1. (**F**) Representation of Ara h VG copy numbers and (**G**) Ara h mRNA expression in liver samples. (**H**) Expression of Ara h proteins in liver and (**I**) plasma. Data are presented as single data points and means ± standard deviation. Statistical testing was conducting using one-way ANOVA statistic test for panels (**B**,**D**–**H**), and Kruskal–Wallis was used for panels (**C**,**I**). * *p* < 0.05, ** *p* < 0.01, *** *p* <0.001 and **** *p* < 0.0001.

With the success of the vector cocktail, we next explored if the hepatic expression of a single peanut allergen could prevent mice from developing a peanut allergy (Figure 4A). Mice transduced with the Ara h1, h2, or h3 expressing vectors developed anaphylaxis, allergic symptoms, IgE, and IgG responses against peanut protein extract that were indistinguishable from control animals (Figure 4B–E). However, the AAV8-Ara-h6 gene transfer was partially successful in preventing peanut allergy. Five of six animals did not experience a change in body temperature, and three mice had substantially improved allergy scores (Figure 4B,C). Three of six mice lacked IgE formation and had markedly lower IgG1 titers (Figure 4D,E). As above, we collected livers at the study endpoint to measure the vector genome copy numbers of the respective vectors, the mRNA levels of the respective Ara h genes, and the protein levels of the respective of the Ara h proteins. Additionally, we measured the respective levels of each Ara h protein in plasma. Vector copy numbers were similar amongst the different treatment groups (Figure 4F). Similar trends were observed between mRNA expression and Ara h protein levels in the liver, as seen with the vector cocktail (Figure 4G,H) and Ara h protein levels in the plasma (Figure 4I). There was a tendency for higher copy numbers and mRNA levels in liver protein extracts when the vector was given alone, compared to when it was given as part of the cocktail (Figure 3F,G versus Figure 4F,G). 

### 3.3. Increased Vector Dose Results in Efficacy for Ara h 3 but Not Ara h1 Expression from Single Vector

**Figure 5:**
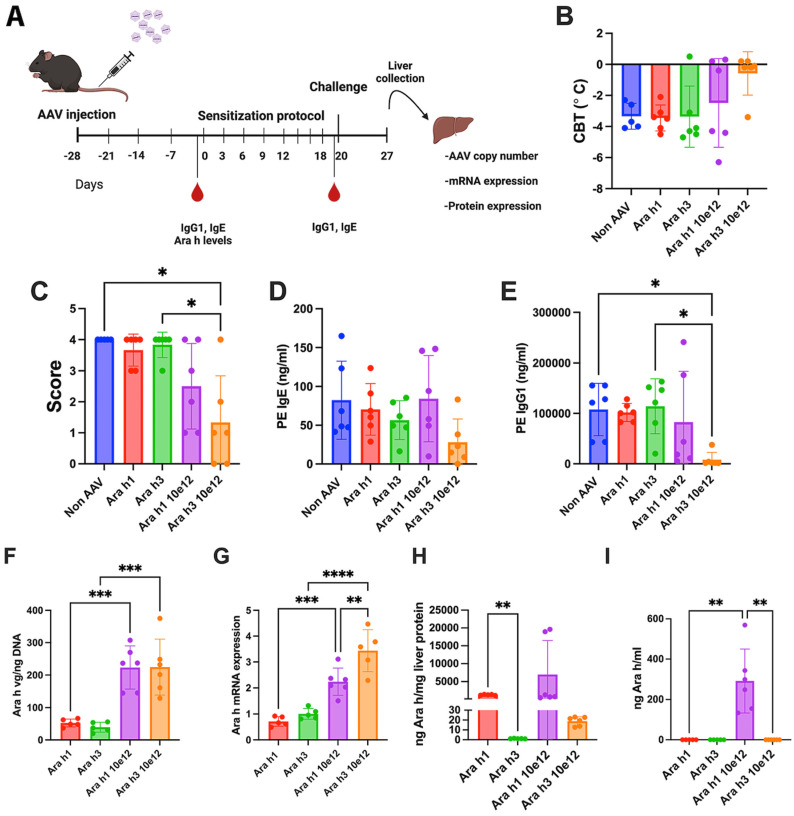
Increased dose of a single AAV-Ara h3 vector prophylactic effect was comparable to the AAV cocktail. (**A**) Experimental timeline followed. (**B**) Changes in core body temperature and (**C**) symptom score (refer to symptom score table here) following challenge with peanut extract. (**D**) Peanut-specific levels of IgE and (**E**) IgG1. (**F**) Representation of Ara h VG copy numbers and (**G**) Ara h mRNA expression in liver samples. (**H**) Expression of Ara h proteins in liver and (**I**) plasma. Data are presented as single data points and means ± standard deviation. Statistical testing was conducting using one-way ANOVA statistic test for panels (**B**,**D**,**F**,**G**), and Kruskal–Wallis test was used for panels (**C**,**E**,**H**,**I**). * *p* < 0.05, ** *p* < 0.01, *** *p* < 0.001 and **** *p* < 0.0001.

Given the absence of Ara h1 and Ara h3 in circulation, we speculated that antigen delivery from hepatocytes to immune cells/draining lymph nodes may also not be efficient. For instance, we previously found that substantially higher vector doses were required to express sufficient levels of cytoplasmic ovalbumin for the induction of ovalbumin-specific Treg compared to secreted ovalbumin [17]. Therefore, we chose to test if we could enhance tolerance induction by using a 10-fold higher dose for these vectors (Figure 5A). In the case of Ara h1, efficacy in the prevention of allergic reactions and antibody formation only marginally improved, with 3 of 6 mice showing a lack of change in body temperature, reduced allergy scores, and IgG formation, while the other 3 mice were comparable to controls (Figure 5B–E). In contrast, increased Ara h3 expression resulted in no change in body temperature, and no or minimal allergy symptoms in 5 of 6 mice, minimal IgE formation, and a lack of IgG formation, with average results approaching those obtained in the mice treated with the vector cocktail (Figure 3B–E and Figure 5B–E). Animals receiving this higher dose had a 3-4-fold increase in hepatic gene copy numbers and mRNA levels (Figure 5F,G). We observed an increase in Ara h1 and Ara h3 protein levels in the liver but not in plasma with the 10-fold increase in vector dose (Figure 5H,I). 

## 4. Discussion

The results from this study further substantiate the emerging body of evidence that liver-derived antigen expression can effectively control pathogenic immune responses in autoimmune and allergy settings, including food allergy. A cocktail of vectors expressing four major peanut allergens were effective in thwarting antibody formation against peanut extracts in mice sensitized by skin exposure, and thus prevented allergic reactions, whereas single vectors expressing either Ara h3 using a high dose (1 × 10^12^ vg) or Ara h6 at the same dose in the cocktail (1 × 10^11^ vg) were partially effective in blunting the allergic reactions. 

Consistent with our earlier results in mice sensitized to chicken ovalbumin, expression levels are critical, so that an increased expression of Ara h3 had a beneficial effect. Since Ara h3 represents approximately 50–60% of the total protein in the peanut extract, an increased expression may have led to enhanced Treg induction and bystander suppression [10]. Expression levels are determined by the combination of AAV serotype/capsid sequence, promoter strength, and vector dose [1,13,57,58]. Here, we used AAV8 because of its high efficacy in gene transfer to murine hepatocytes. Interestingly, despite the use of the same serotype and promoter and only modest differences in gene copy numbers, intrahepatic antigen levels varied substantially between the four protein allergens. Codon-optimization and other modifications may improve the efficacy of these vectors. 

Clinically, patients with peanut allergies can develop IgE specific responses to several peanut proteins, including the four that were selected to express from our AAV vectors. Specific Ara h titers in humans can vary due to several factors, including geographical location, age of exposure, and differences in food processing [39]. In the United States, anti-Ara h2-IgE titers have been shown to be a good predictor for the risk of an allergic response and clinical responses in desensitization trials [40]. Although less abundant than Ara h3 in peanut protein, most IgE reactivity in humans is against Ara h2 and Ara h6 [51,59]. Therefore, our success with Ara h3 in the mouse model may not directly translate to human treatment. The sensitization profiles in humans vary and do not reflect the relative abundance of the different storage proteins in the peanut. A strategy that employs multiple antigens may still provide the best outcomes in patients. Alternatively, one could envision the development of a personalized immunotherapy based on individual sensitization profiles. 

Nonetheless, it is interesting that the adequate expression of a single allergen, such as Ara h3 (and to a lesser extent Ara h6), had strong effect on the allergic phenotype. Future studies should address whether a dominant effect can be achieved over responses to other allergens in the peanut, which may be mediated by active suppression by Treg. In our previous study in a murine model of egg allergy, where we had purified an allergen available for antigen-specific immunoassays, we found that liver-directed AAV gene immunotherapy directed the expansion of allergen-specific FoxP3^+^ Teg and increased the production of regulatory cytokine IL-10 while reducing IgE promoting cytokine IL-13 [13]. Interestingly, among the four peanut protein transgene products, Ara h1 was by far the most highly expressed, but was ineffective in protecting the mouse from allergic reactions to the whole peanut extract when compared to Ara h3. It is possible that, in this mouse model, Ara h1 is less of a contributor to the activation of immune response to whole peanut protein than the other allergens, so that tolerance induction to Ara h1 may not be protective in this model. Ara h3 is most abundant in the peanut extract and may therefore have the most contribution in the sensitization protocol used in the mouse.

Some protein replacement therapies are hampered by allergic reactions to intravenously infused protein drugs. For example, continued injections of coagulation factor IX (FIX) in hemophilia B patients that have developed antibodies against FIX may result in anaphylaxis [60,61]. Similarly, hemophilia B mice on a C3H/HeJ background develop IgE and lethal anaphylactic reactions upon continued IV administration of the human FIX protein [10,62]. Our studies in this model showed that hepatic gene transfer could safely reverse IgG and IgE formation, without allergic reactions to the circulating FIX antigen expressed by hepatocytes [10]. Moreover, mice could then be safely challenged with intravenous FIX, which was lethal in control animals. Our recent study in the egg allergy model also demonstrated the safe reversal of the allergic phenotype in ovalbumin sensitized mice using AAV liver gene transfer [13]. Consistent with these observations, mice sensitized to peanut extract did not show signs of allergy when they were subsequently transduced with the four-vector cocktail. However, since the sensitized control mice did not adequately respond to the delayed intraperitoneal challenge with peanut proteins, we were not able to rigorously test the reversal of a pre-existing peanut allergy by subsequent gene transfer. 

Extensive studies in hemophilia A and B dogs have shown that tolerance induction by liver-directed AAV gene transfer is not limited to rodent models [9,63,64]. Initial evidence for the applicability of this concept to humans is now emerging in a clinical trial on hepatic AAV gene transfer in adult hemophilia A patients with antibodies (“inhibitors”) against factor VIII [11]. Oral and sublingual antigen delivery in patients yields a more durable lack of responsiveness when initiated in young children. While hepatic AAV gene transfer provides immunotherapy in adults, research with liver-directed retroviral gene transfer in neonatal dogs also supports the effectiveness when gene therapy is given early in life [65]. Our recent work also supports that hepatic gene transfer and oral antigen delivery can be combined, with oral delivery inducing LAP^+^ Treg (expressing latency associated peptide of TGF-β on the cell surface) in addition to FoxP3^+^ Treg [21,66]. Furthermore, orally delivered antigen can be found in the liver, suggesting a possible contribution of the liver to oral tolerance induction [62,67].

## 5. Conclusions

In summary, recent studies establish that the tolerogenic capacity of the liver’s immune system is capable of controlling food allergies and allergies against protein therapeutics. Even so, the translation of this concept to human treatment is not straightforward. Current costs for gene therapies are very high, while, for example, plant-based oral antigens could be manufactured at much lower costs [68]. Costs would be a particular challenge if multiple vectors need to be manufactured for a single patient. Experience is still limited regarding the safety of liver gene transfer in humans. Should high vector doses be required, the approach would be less attractive. Our prior studies also show that the balance of the response upon hepatic AAV gene transfer can shift toward immunity to the transgene product, depending on factors such as the levels of expression and the nature of the expressed protein [48,69]. However, in no instance have we observed anaphylaxis related to the AAV hepatic expression of an antigen in mice with pre-existing anti-IgE antibodies to the expressed antigen [10,13]. Further improvement, such as the optimization of codon usage for mammalian cell expression, will likely enable a reduction in vector dose, and alternative expression systems may be developed. In conclusion, the broad applicability of immunotherapy by hepatic gene transfer to gene therapy, anti-drug immune responses, autoimmune diseases, and allergies warrants further pursuit and the optimization of this approach. 

## Figures and Tables

**Table 1:** Allergy symptom score.

Score	Symptom
0	No symptoms
1	Scratching and rubbing around the nose and head
2	Puffiness around the eyes and mouth, diarrhea, piloerection, reduced activity, and/or decreased activity with increased respiratory rate
3	Wheezing, labored respiration, and cyanosis around the mouth and the tail
4	No activity after prodding, or tremor and convulsion
5	Death

## Data Availability

The raw data supporting the conclusions of this article will be made available by the authors on request.

## Supplementary Materials

The following supporting information can be downloaded at: https://www.mdpi.com/article/10.3390/v16071125/s1, Figure S1: Outline of AAV vectors expressing Ara antigens.
