# Supplementary material for: Viral Vector Based Immunotherapy for Peanut Allergy
Source: Viruses. 2024 Jul 13;16(7):1125. doi: 10.3390/v16071125 (PMC11281582; doi:10.3390/v16071125)
Supplement: Supplementary file 1 [file viruses-16-01125-s001.zip › viruses-3086910-supplementary.pdf]

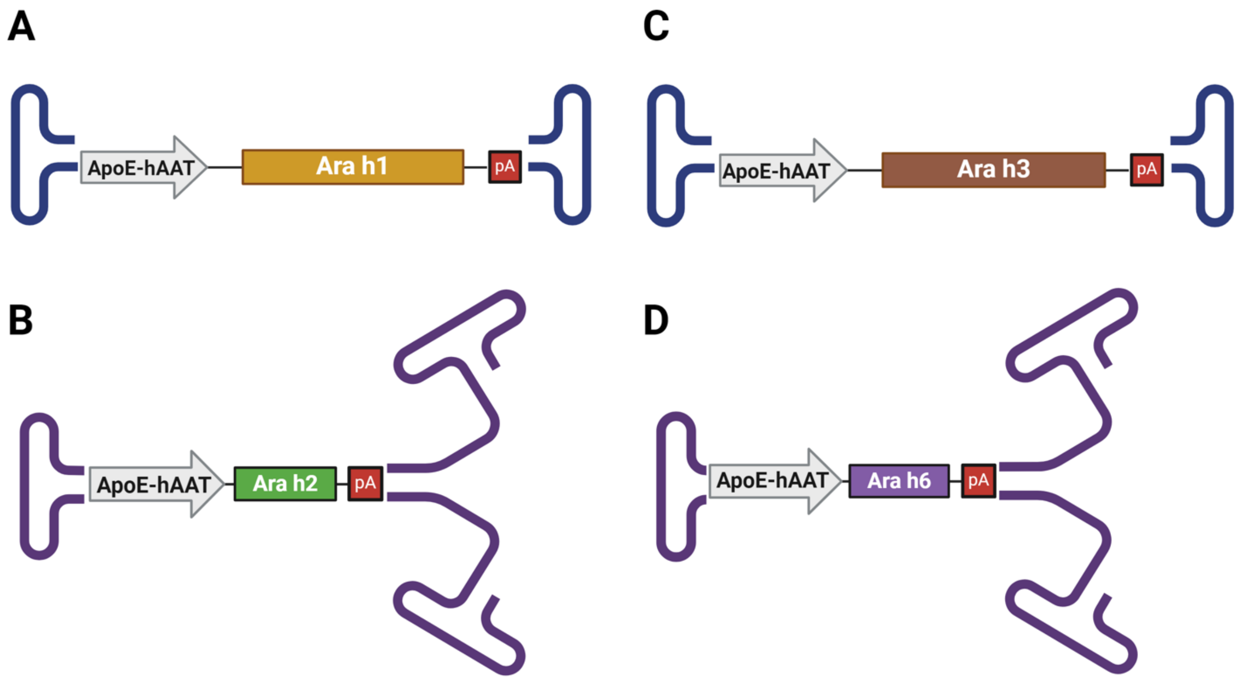

Supplementary Figure S1: Outline of AAV serotype 8 vectors expressing Ara h1 (**A**), Ara h2 (**B**), Ara h3 (**C**), or Ara h6 (**D**) antigens from the hepatocyte-specific enhancer/promoter ApoE/human α1-antitrypsin combination. Vectors for Ara h1 and Ara h3 have self-complementary genomes, while vectors for Ara h2 and Ara h6 have single-stranded genomes.
